# TRIALS AND TRIBULATIONS OF CONDUCTING A GEROSCIENCE TRIAL

**DOI:** 10.1093/geroni/igad104.3673

**Published:** 2023-12-21

**Authors:** Courtney Millar, Kathryn Baldyga, Ikechukwu Iloputaife, Alex Wolfe, Jasmin Kuo, Lewis Lipsitz

**Affiliations:** Hebrew SeniorLife, Marcus Institute, Harvard Medical School, Roslindale, Massachusetts, United States; Hebrew SeniorLife, Marcus Institute, Harvard Medical School, Roslindale, Massachusetts, United States; Hebrew SeniorLife, Marcus Institute, Harvard Medical School, Roslindale, Massachusetts, United States; Hebrew SeniorLife, Marcus Institute, Harvard Medical School, Roslindale, Massachusetts, United States; Tufts University, Medford, Massachusetts, United States; Hebrew SeniorLife, Boston, Massachusetts, United States

## Abstract

Emerging trials of geroscience interventions show great promise in preventing age-related diseases and disabilities. However, there are many challenges that need to be considered when conducting such trials. Our objective is to describe the major challenges when conducting a geroscience intervention, utilizing the STAMINA study as an example. The STAMINA (Senolytics To Alleviate Mobility Issues and Neurological Impairments in Aging) study is a single-arm geroscience-motivated pilot study in which 12 older adults with mild cognitive impairment and slow gait speed are asked to take 12 doses of two senolytic agents (Quercetin and Dasatinib) over 12 weeks. Study challenges were identified and recorded by the study team and categorized into 3 groups: Regulatory, Recruitment, and Unexpected. This is a descriptive analysis and no formal statistical tests were conducted. Eleven major challenges were identified. The Regulatory Challenges included obtaining the IND from the FDA, hiring a clinical research organization (CRO) to monitor compliance with FDA regulations, and establishing FDA compliant data management tools and procedures. The Recruitment Challenges include drug-drug interactions, comorbidities, need for transportation to and from study visits, and fear of drug side effects. Unexpected Challenges included exorbitant drug costs (Dasatinib costs $500 per dose); requirements for drug storage, dispensing, and destruction; multiple safety lab tests; transportation; biomarker processing, transport, and assays; equipment failure; and intercurrent vaccinations and medicines (e.g., COVID-19 and flu vaccines) which may impact biomarker levels. Conducting a geroscience trial has numerous challenges that need to be considered when developing a budget, hiring staff, and assuring compliance.

